# Heterogeneous perception of the ethical legitimacy of unbalanced randomization by institutional review board members: a clinical vignette-based survey

**DOI:** 10.1186/s13063-018-2822-1

**Published:** 2018-08-14

**Authors:** Clarisse Dibao-Dina, Agnès Caille, Bruno Giraudeau

**Affiliations:** 1 Université de Tours, Université de Nantes, INSERM, SPHERE U1246, Tours, France; 2 Université de Tours, Université de Nantes, INSERM, SPHERE U1246, CHRU, CIC 1415, Tours, France; 3 Département Universitaire de Médecine Générale, Faculté de Médecine – Université de Tours, 10 Boulevard Tonnellé, B.P. 3223, 37044 Tours, cedex 1 France

**Keywords:** Unbalanced randomization, Institutional review board, Ethics, Equipoise principle

## Abstract

**Background:**

Institutional review boards must guarantee the ethical acceptability of a randomized controlled trial before it is conducted. However, some may regard an unbalanced randomization ratio as reflecting an absence of uncertainty between the groups being compared. The objective was to assess institutional review board members’ perceptions of whether unbalanced randomization in randomized controlled trials is justified and ethically acceptable.

**Methods:**

Institutional review board members worldwide completed a survey involving clinical vignettes modeling situations classically advocated to explain the use of unbalanced randomization. Institutional review board members were asked whether unbalanced randomization was justified and ethically sound. Answers were collected by using visual analog scales. Data were analyzed by principal component analysis, and a hierarchical ascending classification was created. Verbatim answers were assessed by qualitative content analysis.

**Results:**

We analyzed responses from 148 institutional review board members. Three classes of respondents were identified: class 1 (*n* = 58; 39.2%), mostly skeptics who disagreed with unbalanced randomization, whatever the justification; class 2 (*n* = 46; 31.1%), believers who considered that unbalanced randomization was acceptable whatever the justification, except cost; and class 3 (*n* = 44; 29.7%), circumstantial believers for whom unbalanced randomization may be justified for methodological and safety issues but not cost or ethical issues. When institutional review board members were asked whether unbalanced randomization respected the equipoise principle, the mean quotation was low (4.5 ± 3.3 out of 10), especially for class 1 members.

**Conclusions:**

Institutional review board members perceive unbalanced randomization heterogeneously in terms of its justification and its ethical validity.

**Electronic supplementary material:**

The online version of this article (10.1186/s13063-018-2822-1) contains supplementary material, which is available to authorized users.

## Background

In a recent systematic review of reports of unbalanced randomized controlled trials (RCTs), the prevalence of unbalanced randomization was estimated at 4.7% [1]. Although justifications to use unbalanced randomization were missing in more than 75% of the published reports, the most frequently evoked reason was to obtain more safety data on the experimental treatment, with more patients exposed to it [1, 2]. Among other justifications were considering that it could strengthen the recruitment of patients, expose fewer patients to the potentially inferior treatment, or reduce cost or increase power of per-protocol statistical analyses [1, 2].

Justifying unbalanced randomization should be mandatory, notably because the practice leads to an increased required sample size [3]. Moreover, this design feature could challenge clinical equipoise, with results associated with unbalanced RCTs significantly more often positive than those from matched balanced RCTs [4]. The clinical equipoise principle is an ethical prerequisite for conducting a RCT and is defined by the existence of a genuine uncertainty in the expert medical community about which of the tested treatments will be the most beneficial [5]. Although this ethical principle is usually evoked when considering a specific RCT, it can also be evoked at a meta-level, considering a sample of RCTs. Djulbegovic reported that “there is a predictable relationship between the uncertainty, that is, the moral principle, upon which randomized trials are based, and the ultimate outcomes of randomized trials” [6]. That hypothesis leads to an expectation that over time, we “find no significant difference between the proportion of randomized trials that favor new treatments and those that favor established treatments”, which can be considered the respect of clinical equipoise at a meta-level [6].

Institutional review board (IRB) members have a role in guaranteeing the ethical acceptability of an RCT. Here we investigated their perceptions of whether unbalanced randomization in RCTs is justified and ethically acceptable.

## Methods

### Design

The aim of the study was to assess IRB members’ perceptions of whether unbalanced randomization in randomized controlled trials is justified and ethically acceptable. We used a clinical vignettes-based survey that we administered to IRB members worldwide.

### Selection of IRB members

A list of IRBs was provided by the Director of the Division of Policy and Assurances from the Office for Human Research Protections on April 18, 2012 (available at http://www.hhs.gov/ohrp/). We received names, emails, and addresses of IRB members worldwide.

### Survey questionnaire and administration

We sent the Web-based survey by email via Sphinx© software (SphinxOnline 3.1.5) to IRB members for whom an email address was provided. One reminder was sent to non-respondents at 6 weeks.

The survey included six clinical vignettes (see Additional file 1) to illustrate one of the justifications typically evoked when reporting the summary results of a published unbalanced RCT. The justifications considered were (1) obtaining more safety data, (2) cost, (3) methodological reasons, (4) strengthening patients’ acceptability to participate, (5) high expected drop-out rate, or (6) “bad deal trials” (e.g., when a trial proposes a known inferior treatment option or a treatment with significant harm and no prospect of benefit). IRB members were asked if using an unbalanced randomization was justified and ethically sound in each of these different situations. The survey then explored their ethical perceptions of unbalanced randomization with isolated questions related to the impact on sample size and the equipoise principle. For each question on clinical vignettes or ethical perceptions, members answered by using a visual analog scale (VAS) ranging from 0 (no) to 10 (yes) and were allowed to explain their position in detail. Finally, the survey asked about member characteristics, including age, gender, professional background, experience in planning and approving an unbalanced RCT, and whether they would agree to be recruited as a patient in an RCT with unbalanced randomization.

### Statistical and verbatim analysis

For the questions with a 0–10 VAS response, some questions were re-coded so that we always had a “10, it is acceptable,” or “10, it is ethically sound,” response. Questionnaires with ≥4 of 12 questions with no answer were discarded. Otherwise, we imputed a value derived from an ad hoc regression model fitted by using a complete case. To identify clusters of IRB members who differed regarding their perceptions of unbalanced randomization, we used principal component analysis followed by an ascending hierarchical clustering analysis. Finally, verbatim transcripts were evaluated by qualitative content analysis [7]. After reading the entire verbatim text, the text was divided into meaningful units, which were then coded and classified into larger topics.

## Results

### Global description of the respondents and their answers

**Fig. 1:**
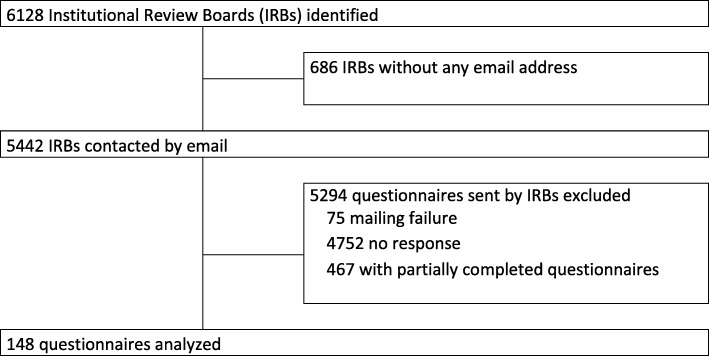
Flowchart

We analyzed 148 questionnaires (Fig. 1). The responders’ characteristics are in given in Table 1.

**Table 1:** General characteristics of institutional review board (IRB) members by class of perception of unbalanced randomization in randomized controlled trials (RCTs)

Characteristics	Total (*n* = 148)	Class 1^b^ (*n* = 58)	Class 2 (*n* = 46)	Class 3 (*n* = 44)
Age, years, mean ± standard deviation	51 ± 11	50 ± 12	54 ± 10	50 ± 12
Gender male	81 (55.5)	37 (64.9)	26 (57.8)	18 (41.9)
Professional background				
Medical/surgical physician	48 (32.7)	23 (40.3)	13 (28.9)	12 (27.3)
Statistician/epidemiologist	20 (13.6)	7 (12.3)	7 (15.6)	6 (13.6)
Philosopher/ethicist	13 (8.8)	4 (7.0)	2 (4.4)	7 (15.9)
Other ^a^	66 (44.9)	23 (40.3)	23 (51.1)	19 (42.2)
Involved in:				
Planning an RCT	93 (62.8)	39 (67.2)	29 (63.0)	25 (56.8)
Planning an unbalanced RCT	30 (34.9)	7 (18.4)	16 (55.2)	7 (31.8)*
Approving an unbalanced RCT	74 (54.0)	26 (45.6)	24 (53.3)	24 (54.6)
Unbalanced randomization raises problems	19 (27.1)	13 (50.0)	3 (12.5)	3 (12.5)*
Agree to participate in an unbalanced RCT	63 (81.8)	15 (25.9)	25 (54.4)	23 (52.3)*

Data are no. (%) unless indicated

*P < 0.05

^a^Other professional backgrounds included nurse (*n* = 7), pharmaceutical scientist (*n* = 7), sociologist (*n* = 6), psychologist (*n* = 5), biologists/chemist (*n* = 4), educational researcher (*n* = 3), administrator (*n* = 3), physiologist (*n* = 2), lawyer (*n* = 2), engineer (*n* = 2), alternative medicine (*n* = 1), genetics (*n* = 1), physicist (*n* = 1), other imprecise backgrounds (“researcher” or “IRB member”, *n* = 17), and missing data (*n* = 5)

^b^Class 1 = skeptics in the ethical justification of using an unbalanced randomization, whatever the reason

Class 2 = believers in the ethical justification of using an unbalanced randomization, whatever the reason

Class 3 = circumstantial believers evoking ethical and cost issues rather than methodological ones

Their professional background was heterogeneous; one third (32.7%) of them were medical/surgical physicians, but statisticians, epidemiologists, and philosophers were also represented. One third of respondents (34.9%) had been involved in planning an unbalanced RCT, and 54.4% had been involved in approving the conduct of an unbalanced RCT as an IRB member.

The IRB members’ perceptions of unbalanced randomization are provided in Table 2.

**Table 2:** IRB members’ opinions of justifications for unbalanced randomization by class of perception of unbalanced randomization

Justifications for unbalanced randomization^a^	Total (*n* = 148)	Class 1^b^ (*n* = 58)	Class 2 (*n* = 46)	Class 3 (*n* = 44)
Gaining experience with the treatment				
Justification considered acceptable	6.1 ± 3.3	3.2 ± 2.6	7.5 ± 2.6	8.2 ± 1.9
Justification considered ethically sound	6.0 ± 3.4	4.1 ± 3.4	7.0 ± 3.0	7.5 ± 2.3
Cost issues				
Justification considered acceptable	3.2 ± 3.0	1.9 ± 1.6	4.8 ± 3.5	3.3 ± 3.2
Justification considered ethically sound	3.1 ± 3.3	1.8 ± 2.2	3.9 ± 3.5	4.0 ± 3.6
Increasing patient acceptability				
Justification considered acceptable	5.4 ± 3.4	4.4 ± 3.2	7.5 ± 2.7	4.6 ± 3.3
Justification considered ethically sound	5.2 ± 3.5	4.5 ± 3.5	6.7 ± 3.1	4.6 ± 3.4
Reduction in expected dropout				
Justification considered acceptable	5.7 ± 3.3	4.7 ± 3.4	7.0 ± 2.8	5.7 ± 3.3
Justification considered ethically sound	6.0 ± 3.3	4.8 ± 3.4	7.6 ± 2.2	5.8 ± 3.4
Ethics issues (e.g., bad deal trial)				
Justification considered acceptable	3.6 ± 3.4	2.5 ± 2.5	6.4 ± 3.4	2.0 ± 2.1
Justification considered ethically sound	2.7 ± 3.5	1.3 ± 2.2	5.8 ± 3.5	1.4 ± 2.6
Methodological reasons				
Justification considered acceptable	6.0 ± 3.3	5.1 ± 3.3	6.7 ± 3.2	6.4 ± 3.2
Justification considered ethically sound	5.7 ± 3.4	4.9 ± 3.5	6.1 ± 3.3	6.3 ± 3.1

Data are mean ± standard deviation (SD) on a visual analog scale (0–10)

^a^The mean score represents the opinion of IRB members from 0, totally unjustified or unethical, to 10, totally justified or ethical

^b^Class 1 = skeptics in the ethical justification of using unbalanced randomization, whatever the reason

Class 2 = believers in the ethical justification of using unbalanced randomization, whatever the reason

Class 3 = circumstantial believers evoking ethical and cost issues rather than methodological ones

The most ethically sound justifications were gaining experience in the treatment (mean VAS, 6.0 ± 3.4) and reduction in expected drop-outs (6.0 ± 3.3), whereas the most ethically unsound justifications were to decrease the study cost (3.1 ± 3.3) and resolve ethical issues (2.7 ± 3.5).

### Hierarchical ascending classification

We retained three classes of respondents from the hierarchical ascendant classification (Table 2). According to the mean response profile for each class, we characterized respondents as follows: class 1 (*n* = 58, 39.2%) represented “skeptics” who disagreed with the use of unbalanced randomization, class 2 (*n* = 46, 31.1%) represented “believers” who agreed with the use of unbalanced randomization for whatever reason (except cost issues), and class 3 (*n* = 44, 29.7%) also represented believers but could be considered “circumstantial believers” because they agreed with the use of unbalanced randomization mainly for safety and methodological motivations but not ethical ones.

The characteristics of the IRB members in the three classes are listed in Table 1. Class 1 mainly contained medical/surgical physicians and class 3, mainly philosophers/ethicists; statisticians/epidemiologists were equally distributed among the three classes. Significantly more class 2 members had been involved in planning an unbalanced RCT as compared with the other two classes (*P* = 0.013). Half of class 1 members considered that unbalanced randomization raises problems, as compared to 12.5% of class 2 and 3 members (*P* = 0.008). Finally, about one quarter of class 1 members would agree to be recruited in an unbalanced RCT as compared to about half of class 2 and 3 members (*P* = 0.007).

### Ethical considerations of unbalanced randomization

The responses to the questions on ethical considerations of unbalanced randomization are given in Table 3.

**Table 3:** Responses for the ethical considerations of unbalanced randomization by class of perception of unbalanced randomization

Ethical considerations of unbalanced randomization^a^	Total (*n* = 148)	Class 1^b^ (*n* = 58)	Class 2 (*n* = 46)	Class 3 (*n* = 44)
Increase in sample size is ethically acceptable	6.1 ± 3.1	5.2 ± 2.8	7.2 ± 2.7	5.9 ± 3.4*
Non-inferiority trials raise distinct issues regarding unbalanced randomization	5.6 ± 3.0	5.4 ± 2.9	5.6 ± 3.0	5.8 ± 3.0
Unbalanced randomization respects equipoise	4.5 ± 3.3	3.6 ± 2.9	5.5 ± 3.5	4.8 ± 3.2*
Beginning a trial with previous negative and positive trial results in equal proportion is ethical	6.2 ± 2.8	6.2 ± 2.6	6.8 ± 3.1	5.4 ± 2.8
Equipoise exists with an equal proportion of negative and positive trials before beginning a new trial	6.0 ± 3.2	5.5 ± 3.3	6.9 ± 3.2	5.8 ± 3.0

Data are mean ± standard deviation (SD) by visual analog scale (0–10)

*P < 0.05

^a^The mean score represents the opinion of IRB members from 0, total disagreement, to 10, total agreement

^b^Class 1 = skeptics in the ethical justification of using unbalanced randomization, whatever the reason

Class 2 = believers in the ethical justification of using unbalanced randomization, whatever the reason

Class 3 = circumstantial believers evoking ethical and cost issues rather than methodological ones

Class 2 members more frequently considered that the increase in sample size was ethical than did class 1 and 3 members (*P* = 0.005). Class 1 members less frequently considered that unbalanced randomization did not respect equipoise than did class 2 and 3 members (*P* = 0.012). The classes did not differ in the other ethical considerations of unbalanced randomization.

### Verbatim analyses

#### Justifications for unbalanced randomization

Among the 38 respondents who considered that obtaining more safety data can be considered as a non-problematic justification for unbalanced randomization, 7 advocated it as long as participants were well informed about the study and agreed to participate with informed consent (*It’s important to obtain more safety data. The consent form must clearly inform patients of the unbalanced randomization.*) Conversely, cost issues were seen as insufficient to justify unbalanced randomization for 102 respondents because decreasing the sample size with a balanced randomization can be an alternative (*Why not enroll fewer patients overall?*). Otherwise, 10 respondents considered that unbalancing randomization to increase patient recruitment or for easier recruitment can induce bias in the study results (especially for patient-reported outcomes) because patients can indeed think that one treatment is better than another, which can then influence their assessment (*Presumably, the population [is] convinced that supportive care is good, which is why it is unbalanced in that direction. Would the study be able to cope with such a preconceived perception?*). Three class 2 respondents (“believers”) also considered that if drop-outs are expected in one group, using an unbalanced randomization to recruit more patients in this latter group would lead to balanced groups in a per-protocol analysis (*[With] the equivalence approach, the unbalanced randomisation obviously minimises the sample size for the per-protocol analysis and thus is acceptable too.*). A majority of respondents (*n* = 109) agreed that unbalancing randomization because one of the treatments is known to be inferior is not justified because such a situation is unethical (*If it is already known to be “inferior”, [then] it is not in clinical equipoise and may not warrant the trial.*). Indeed when a trial’s response is a priori known, the equipoise principle is violated, and 109 responders considered that the trial must not be performed. Nevertheless, some respondents, all from class 2, considered that such trials can be justified to obtain drug approval (*n* = 5) (*I think that trials are needed to the agency regulatory to accept a new drug. If there are information about the more benefit of one of the alternatives, I think that is too justified to use unbalanced randomization to achieve the results before and get the authorization for used it. Maybe it’s not ethical not use it!*) or in case of bad deal trials in case of life-threatening conditions (*n* = 3). They specified that in such situations, the minimal requirements are to adequately inform patients, to be scientifically rigorous, and to minimize patient harms. Seventeen respondents agreed with the use of unbalanced randomization for methodological reasons only if it is statistically and scientifically required and justified (*I believe as long as there is an appropriate statistical analysis and informed consent, I could live with this.*). Finally, some respondents evoked some further justification for unbalanced randomization such as early-phase studies (*n* = 2), with restricted availability of a drug (*n* = 2), to have enough power for subgroup comparison (*n* = 2), or in situations with limited risk of harm (*n* = 21).

#### Ethical considerations for unbalanced randomization

Seventy three respondents considered that unbalanced randomization does not respect the equipoise principle and that only balanced randomization respects it *(Almost by definition, unequal randomization indicates a bias that one treatment is better than another.)*. For 26 respondents, the randomization ratio did not need to be a direct reflection of that uncertainty *(I think they are unrelated. Randomization is a method to control bias for me. The way we use it is the issue I have to look into. It has nothing to do whether the treatment will provide [an] effect or not in the general context of equipoise.).*

## Discussion

IRB members’ perceptions of the ethical legitimacy of unbalanced randomization in RCTs was heterogeneous: some (*n* = 46) were believers, whatever the justification; others (*n* = 44) were circumstantial believers only for methodological or safety justifications; and others (*n* = 58) were skeptics, whatever the justification.

Physicians were mainly skeptics (*n* = 23/48) about the legitimacy of unbalanced randomization, especially for cost and ethical issues. This observation may be related to their duty of care for patients. They face a dilemma: to improve scientific knowledge and to provide the best care to their patients at the same time. This duty of care certainly overlapped cost justifications to use unbalanced randomization, because they mainly considered unbalanced randomization unethical as skeptics. Some authors considered cost reasons to use an unbalanced randomization as acceptable if it is the only way to perform an essential trial [8, 9]. However, unbalanced randomization usually allocates more patients to the intervention group when the control group almost always incurs lesser cost [8].

Ethicists and philosophers were mostly circumstantial believers of unbalanced randomization for safety and methodological reasons only if it was scientifically required (*n* = 7/13). However, they detailed their opposition to the use of an unbalanced randomization for cost or ethical reasons. We could consider that they disagreed with ethical and cost justifications and agreed with methodological and safety reasons under a scientific caution. However, methodological justifications are numerous and not often effective. For instance, we ignore the situation where the practice, when using an unbalanced randomization, effectively decreases patient withdrawals [8]. The use of unbalanced randomization could even lead to a “therapeutic mis-estimation,” whereby patients enrolled in a trial in which they have more chances to obtain the intervention treatment could have false expectations about its efficacy [8]. For instance, in a trial in which patients had eight times more chances to be allocated to a treatment for migraine than placebo, the placebo response was greater than in other balanced randomized trials [10]. Therefore, the unbalanced randomization could lead to biased results.

Unbalanced randomization justified in terms of safety could be useful when safety signals are vital to know and easily attributable to the intervention [8]. However, the interpretation of safety data is not easy, and this justification is less valid when the experience with the treatment increases [8]. Furthermore, safety data are not well reported even when they constitute the principal justification for using unbalanced randomization [1].

Three IRB members found it ethical to expose patients to a more risky trial if they were in a life-threatening situation and only if there was a scientific justification or certainty in the efficacy or absence of harms of the new treatment. However, uncertainty is required to begin a controlled trial [11], so these conditions are unethical. The results of Table 3 emphasized that responders agreed with the requirement of the equipoise principle to be respected before conducting a trial. They mostly considered that unbalanced randomization did not respect the equipoise principle (*n* = 73), which leads to a concern about the ethical legitimacy of such a design. Some others (*n* = 26) considered that the randomization ratio did not necessarily have to reflect the uncertainty between the treatments to be tested as long as the equipoise principle was respected. This may refer to respecting the clinical equipoise, which is a “genuine uncertainty within the expert medical community about the preferred treatment” as defined by Freedman [5] and which is mandatory before conducting a trial. As long as this uncertainty among the expert medical community is respected, this supposes that the exact proportion of randomization may vary, as described for response adaptive randomization designs [12].

This is the first study exploring IRB members’ perceptions about the ethical legitimacy of using unbalanced randomization in a trial. Some limitations of our study include the limited number of responses (148 responses from 5442 contacted IRB members) and the risk of IRB members misunderstanding the definition of the equipoise principle. The equipoise principle that we referred to in our study was the clinical equipoise as defined by Freedman [5]. However, different types of equipoise exist depending on which person — patient (patient’s equipoise) or clinician (theoretical equipoise) — or which group of patients (community equipoise) or clinician experts (clinical equipoise) was/were in a state of uncertainty [13]. These different types of equipoise were not clearly described in the different sources that we gave to IRB members (see Additional file 1) [14, 15]. Avins’ reference explored the ethical dilemma between respecting theoretical equipoise and clinical equipoise in trials with unbalanced randomization [14]. The Cochrane collaboration definition of equipoise was easily available to everyone, and it may have been confusing because it dealt with a person without specifying who this person was (the patient or the clinician) or whether it could also imply a group of persons (patients or clinical experts) [15]. Hence, this Cochrane definition can be interpreted as the theoretical equipoise definition rather than the clinical equipoise definition. Even if some responders clearly referred to clinical equipoise in their comments (*If it is already known to be “inferior”, [then] it is not in clinical equipoise and may not warrant the trial.*), we cannot exclude a misunderstanding of theoretical and clinical equipoise by some responders. This lack of clarity may be explained by the existing debate about which kind of equipoise must be prioritized [13]. The debate goes even further, with authors questioning the relevance of the equipoise principle [16], as illustrated by the comments of class 2 responders about trials for obtaining drug approval. Some authors claim that the clinical equipoise defined by Freedman [5] (i.e., uncertainty among the clinical experts community) is relevant as an ethical prerequisite for conducting randomized trials because it “ensures that they can participate without having to worry that their interests are being sacrificed at the altar of science” [16]. However, other authors claim that the equipoise principle limits the conduct of trials of scientific interest and that other frameworks such as the net risks framework may be sufficient to reduce the risk of participants participating [16]. Even if this debate is still ongoing, IRB members must ensure that the rights of humans participating as subjects in a research study are respected before deciding whether a trial can be conducted. One study investigated factors affecting IRB members’ decisions to approve or not approve clinical studies among 42 institutions with 208 participants [17]. The perceived uncertainty between benefits and harms of proposed treatments to be compared was the main factor affecting IRB members’ decisions to approve a trial’s protocol, before adherence to the research aim, potential harms, and study design [17].

## Conclusions

There was no consensus among IRB members in their perception of the ethical legitimacy of unbalanced randomization. They typically considered justifications advocated by authors who used unbalanced randomization in trials as flawed. The use of unbalanced randomization in trials must be questioned.

## Additional file

Additional file 1: Survey sent to institutional review board (IRB) members with clinical vignettes illustrating classically evoked justifications of unbalanced randomization. (DOC 59 kb)

## Abbreviations

IRB: Institutional review board
RCT: Randomized controlled trial
VAS: Visual analog scale
