# Supplementary material for: Heterogeneous perception of the ethical legitimacy of unbalanced randomization by institutional review board members: a clinical vignette-based survey
Source: Trials. 2018 Aug 14;19:440. doi: 10.1186/s13063-018-2822-1 (PMC6092831; doi:10.1186/s13063-018-2822-1)
Supplement: Supplementary file 1 — Survey sent to institutional review board (IRB) members with clinical vignettes illustrating classically evoked justifications of unbalanced randomization. (DOC 59 kb) [file 13063_2018_2822_MOESM1_ESM.doc]

Appendix 1. Survey sent to Institutional Review Board (IRB) members with clinical vignettes illustrating classically evoked justifications of unbalanced randomization.

EMAIL INVITATION

Research project on unbalanced randomization and equipoise

Dear colleague, I am conducting a research project on unbalanced randomization and ethics in randomized controlled trials, especially whether such trials respect the principle of “equipoise.” This project has been approved by the Institutional Review Board (IRB) of Paris North Hospital (project n° 11-118) and is supported by a grant from the French Ministry of Health (PHRC 2011 n°10-1). I am interested in the opinion of IRB members on this question of unbalanced randomization and ethics. Therefore, I would very much appreciate receiving your answers to my questions by clicking the following link: XXX. The protocol of my project is summarized at the beginning of the questionnaire. I would be pleased to provide you any further information you may require and thank you in advance for your help”. We also joined the summary of the protocol as follows:

Summary of the protocol:

Context: Unbalanced randomization is not a frequently used design, especially because of the high number of participants required^1^. Nevertheless, some authors encourage the use of unbalanced randomization. Pocock emphasized the potential ethical advantages of unbalanced randomization because more subjects could be randomized to the arm thought to be superior^2^. According to Avins, unbalanced randomization could be a way to “reconcile the ethical mandate and the practical reality (…) in the absence of true equipoise”^3^.

Objective: To determine whether unbalanced randomization is an ethical design and respects the state of equipoise.

Methods: The work will be divided into 3 parts

- A review of the literature. Reports of trials with unbalanced randomization ratios will be selected from Medline via PubMed with limits to “core clinical journals”, “randomized controlled trials” and articles published in 2009 or 2010. The selected articles will be systematically reviewed with use of a standardized form exploring the funding source, randomization, safety data and sample size calculation.

- A survey of main investigators. The main investigator/corresponding author of the selected articles will be contacted to collect details about the reasons for choosing such a design.

- A survey of IRB's members opinion. IRB's members will be contacted to give their opinion on unbalanced randomization design.

Endpoints: Reasons for using an unbalanced randomization; special ethical features concerning this design.

Expected consequences: To conclude whether unbalanced randomization is an ethical design.

References:

1. Dumville JC, Hahn S, Miles JNV, Torgerson DJ. The use of unequal randomisation ratios in clinical trials: a review. Contemp Clin Trials 2006;27(1):1-12.

2. Pocock SJ. Clinical trials: a practical approach. Chichester, John Wiley and Sons; 1995.

3. Avins AL. Can unequal be more fair? Ethics, subject allocation, and randomised clinical trials. J Med Ethics 1998;24:401-8.

CLINICAL VIGNETTES

REASONS FOR USING UNEQUAL RANDOMIZATION RATIOS

Authors justify their choice for unequal randomization with several reasons. We consider each reason one by one and ask for your opinion by choosing one of 2 answers. The end of the questionnaire asks a few questions about you and your experience in this area.

UNEQUAL RANDOMIZATION AND GAINING EXPERIENCE WITH THE TREATMENT

Some authors justify their choice of unbalanced randomization to gain experience or obtain more safety data on the new drug^1^. Example: comparison of effect of “combination of new chemotherapy agents” and “standard chemotherapy” in patients with advanced gastric cancer on overall response rate. Patients were randomized in a 2:1 ratio (intervention:control) to obtain additional safety data about the “combination of new chemotherapy agents” (Cocconi G. J Clin Oncol 1994;12:2687–93). In this case, do you think that using an unbalanced randomization is justified?

No Yes

Is this use of unbalanced randomization ethically sound for you?

No Yes

Could you explain your answer or position in detail?

UNEQUAL RANDOMIZATION AND COST

Some authors justify their choice of unbalanced randomization for cost reasons (e.g., when one of the treatment arms is expensive, especially the interventional treatment).^1^ Example: comparison of effect of simvastatine and placebo on coronary heart disease. If patients were randomized in a 1:2 ratio (intervention:control), the trial cost would have been £2 993 101 instead of £4 448 641 for a 1:1 ratio (Torgerson D. J Health Serv Res Policy 1997;2:81– 5).

In this case, do you think that using unbalanced randomization would have been justified?

No Yes

Is this use of unbalanced randomization ethically sound for you?

No Yes

Could you explain your answer or position in detail?

UNEQUAL RANDOMIZATION AND PATIENT ACCEPTABILITY

Other authors justify their choice of unbalanced randomization as increasing recruitment for the study^1^. Example: comparison of effect of a supportive-expressive therapy and usual care on survival for women with metastatic breast cancer. Patients were randomized at a 2:1 ratio (intervention:control) to increase patients’ agreeing to participate (Goodwin PJ. N Engl J Med 2001;345:1719– 26).

In this case, do you think that using unbalanced randomization is justified?

No Yes

Is this use of unbalanced randomization ethically sound for you?

No Yes

Could you explain your answer or position in detail?

UNEQUAL RANDOMIZATION AND EXPECTED DROPOUT

Other authors justify their choice of unbalanced randomization as decreasing the rate of lost to follow-up^1^. Example: comparison of effect of a dietary intervention and a knee strengthening exercise on knee pain reduction and knee function improvement in overweight and obese adults. Patients were randomized at a 3:2 ratio (dietary:exercise) because the investigators expected that more people would withdraw from the dietary intervention group than the exercise group (Jenkinson CM. BMJ 2009;339:b3170).

In this case, do you think that using unbalanced randomization is justified?

No Yes

Is this use of unbalanced randomization ethically sound for you?

No Yes

Could you explain your answer or position in detail?

UNEQUAL RANDOMIZATION AND ETHICS

Some authors justify their choice of unbalanced randomization by ethics, especially for “bad-deal trials”. A bad-deal trial is a clinical trial “in which the medical community knows that an option offered in the trial is significantly inferior to alternative available treatment modalities, or that an option offered in the trial would pose a non-negligible risk of significant harm with no prospect for significant benefit, even if no alternative treatment modality has proven effective” (Jansen LA. HAsong Cent Rep 2005;35(5):29-36). For example, a bad-deal trial would compare sertraline versus placebo (with a 2:1 randomization ratio) in patients with depression when we know in advance the inferiority of the placebo.

In these cases, do you think that using unbalanced randomization is justified?

No Yes

Is this use of unbalanced randomization ethically sound for you?
No Yes

Could you explain your answer or position in detail?

UNEQUAL RANDOMIZATION AND METHODOLOGICAL REASONS

Other authors justify their choice of unbalanced randomization by methodological aspects of the study (to increase power, improve the estimation of the outcome measure, etc.) ^1^. Example: comparison of effect of an implantable defibrillator with conventional medical therapy on death rate in patients with reduced left ventricular function. Patients were randomized at a 3:2 ratio (intervention:control) to increase the power of a secondary analysis (survival curves).

In this case, do you think that using unbalanced randomization is justified?

No Yes

Is this use of unbalanced randomization ethically sound for you?

No Yes

Could you explain your answer or position in detail?

Do you think that there are other situations in which an unequal randomization ratio could be justified in a trial?

Yes

No

Do not know

If yes, could you define what type of situation(s):   FORMTEXT      

# UNBALANCED RANDOMIZATION AND ETHICS

AT THE LEVEL OF A UNIQUE TRIAL

Using unbalanced randomization ratios increases the required sample size of patients. Do you think that this is ethically acceptable?

No Yes

Could you explain your answer or position in detail?

Do you think that using unbalanced randomization raises distinct issues in non-inferiority trials and superiority trials?

Identical issues Different issues

Could you explain your answer or position in detail?

The Cochrane collaboration defines equipoise as “a state of uncertainty where a person believes it is equally likely that either of two treatment options is better” ([www.cochrane.org](http://www.cochrane.org/)).

Do you think that using unbalanced randomization respects the equipoise principle?

No Yes

Could you explain your answer or position in detail?

AT THE LEVEL OF ALL TRIALS

Djulbegovic wrote that “equipoise exists if the number of studies in which new therapies are preferred (…) over standard therapies (…) is similar” (Djulbegovic B. Lancet 2000;356:635-8). Do you think that beginning a trial with previous negative and positive trial results in an equal proportion is ethical?

No (not ethical) Yes (ethical)

Could you explain your answer or position in detail?

In this case, do we consider that equipoise exists when there are an equal proportion of negative and positive trial results before beginning a trial?

No (no equipoise) Yes (equipoise OK)

Could you explain your answer or position in detail?

ABOUT YOURSELF

Age:

Sex:

Male

Female

Professional background:

Medical/surgical physician

Statistician/epidemiologist

Philosopher/ethicist

Other:

Were you ever involved in planning a randomized controlled trial?

Yes

No

Don’t remember

If yes, were you ever involved in planning a randomized controlled trial with unbalanced randomization?

Yes

No

Don’t remember

As an IRB member, have you ever been involved in approving an unequal randomized controlled trial?

Yes

No

Don’t remember

If yes, did the situation raise problems?

Yes

No

Don’t remember

If you were asked to participate in a trial with an unbalanced randomization as a patient, would you agree to participate?

Yes

No

Do not know

Could you explain your answer or position in detail?
